# HIV and Diarrhea: Clinical Characteristics and Etiology at a High‐Complexity Center in Southwest Colombia

**DOI:** 10.1155/arat/7874615

**Published:** 2026-06-23

**Authors:** Carlos Arturo Rojas-Rodríguez, Juan David Sossa-Camacho, Leidy Johanna Hurtado-Bermúdez, Juan Camilo Ramírez-Márquez, Edgar David Salazar-Cardona, Daniela Piedrahita-García, Álvaro José Martínez-Valencia, Tatyana Saa-Henao, Eugenia Flórez-Jordan, Carlos Julio Vargas-Potes

**Affiliations:** ^1^ School of Health Sciences, Universidad Icesi, Cali, Colombia, icesi.edu.co; ^2^ Internal Medicine Unit, Department of Gastroenterology, Fundación Valle Del Lili, Cali, Colombia, valledellili.org; ^3^ Clinical Research Center, Fundación Valle del Lili, Cali, Colombia, valledellili.org; ^4^ Internal Medicine Unit, Department of Infectious Diseases, Fundación Valle del Lili, Cali, Colombia, valledellili.org; ^5^ Internal Medicine Unit, Fundación Valle del Lili, Cali, Colombia, valledellili.org

**Keywords:** etiology, antiretroviral therapy, diarrhea, HIV

## Abstract

**Objective:**

Human immunodeficiency virus (HIV) infection remains highly prevalent and represents a significant economic burden on healthcare systems. The World Health Organization (WHO) reported 39 million people living with HIV (PLWH) in 2023. In Colombia, 185,954 prevalent cases were reported during 2024. Diarrhea affects up to 50% of PLWH. This study aimed primarily to estimate the prevalence of infectious versus noninfectious etiology of diarrhea among adults with HIV and to compare immunological and clinical events.

**Design/Method:**

Cross‐sectional observational study included adult patients with HIV and diarrhea who received care at Fundación Valle del Lili between 2014 and 2022. Clinical variables, diarrhea etiology, treatment, and clinical events were analyzed according to the chronologic classification of diarrhea using chi‐squared and F tests for qualitative variables and Kruskal–Wallis and ANOVA tests for quantitative variables. Effect sizes are reported as odds ratios (ORs) with 95% confidence intervals (CIs) for binary outcomes between infectious and noninfectious diarrhea. Limitations include the cross‐sectional design, single‐center setting, and convenience sampling.

**Results:**

Among the patients, 73.8% were male, and the mean age was 42 years. De novo diagnosis was made in 26.2% and 90.5% of previously diagnosed individuals receiving antiretroviral therapy. The 23% had AIDS‐defining conditions. One‐third of the patients had acute diarrhea. Infectious etiology was found in 65.5% of patients and was associated with higher viral loads and lower CD4 (+) T‐cell counts compared to noninfectious diarrhea. Antibiotic therapy was used in 57%, and diarrhea had resolved in 77/84 (91.7%) by discharge. Associated mortality was 2.8%.

**Conclusions:**

The predominant cause of diarrhea was infectious. Infectious etiology and persistent diarrhea were associated with higher viral loads and lower CD4 (+) T‐cell counts, which may be linked to poorer clinical outcomes in HIV‐infected patients. Further studies are required to better understand these relationships.

## 1. Introduction

Despite advances in the prevention and management of human immunodeficiency virus (HIV) infection, the disease continues to have a high prevalence and represents a significant economic burden. According to the World Health Organization (WHO), by the end of 2023, there were 39 million people living with HIV (PLWH) worldwide, with an annual mortality rate of 1.6% among the infected population [1]. In Colombia, a total of 185,954 prevalent cases of HIV infection were reported during 2024 [1].

Diarrhea remains as one of the main determinants of morbidity and mortality in PLWH, affecting up to 50% of this population, with a significant impact on their quality of life at both social and occupational levels. In addition, it imposes high costs on the healthcare system [2].

With the introduction of antiretroviral therapy (ART) in the 1990s, a transformation in the etiological spectrum of diarrhea was observed, shifting from infectious to noninfectious causes. For instance, a study by Call et al. published in 2000 documented a reduction in infectious causes from 53% to 13%, with a simultaneous increase in noninfectious causes from 32% to 71% [3]. Several studies have shown that infectious causes tend to occur in PLWH with CD4 (+) T‐cell counts below 200 cells per cubic millimeter, while noninfectious etiologies are associated with HIV enteropathy, ART‐related adverse effects, HIV‐related neoplasms, autonomic neuropathy, and chronic pancreatitis [2, 4].

This study aimed primarily to estimate the prevalence of infectious versus noninfectious etiology of diarrhea among adults with HIV and to compare immunological (CD4 and VL) and clinical events (hospital stay, intensive care unit [ICU] need, and death) between etiological groups.

## 2. Materials and Methods

### 2.1. Study Design, Population, and Selection Criteria

A cross‐sectional observational study was conducted, including adult patients over 18 years of age of both sexes with a confirmed diagnosis of HIV infection who presented with diarrhea as a primary or secondary symptom upon admission. These patients were treated in the emergency department, inpatient wards, or ICU of the hospital between January 1, 2014, and December 31, 2022. Patient selection was based on ICD‐10 diagnostic codes for HIV infection, in combination with codes related to gastroenteritis or colitis. In total, 121 patients were initially identified. Patients with incomplete information or those who did not present with any episodes of diarrhea upon clinical record review were excluded. After applying the eligibility criteria, a final sample of 84 patients was obtained.

### 2.2. Sample Size

No a priori sample size estimation was performed, as this was a retrospective study based on convenience sampling of all eligible patients during the study period. In line with current statistical recommendations, post hoc power calculations were not conducted, as they are considered uninformative. Instead, results are presented with effect sizes and 95% confidence intervals (CIs) to allow interpretation of the magnitude and precision of the observed associations.

### 2.3. Data Collection and Management

All clinical, demographic, and laboratory data were obtained from patients’ medical records. Microbiological data were extracted directly from the institutional laboratory reports and stored in an electronic database managed by the institution’s clinical research center. Patient identity was protected through coded records and restricted access to the investigators.

### 2.4. Variables

This study included clinical, etiological, treatment‐related, and clinical events variables (Supporting file l).

### 2.5. Laboratory Methods

Multiplex PCR: Etiology of infectious diarrhea was investigated using the BioFire FilmArray Gastrointestinal (GI) Panel (bioMérieux), a syndromic PCR multiplex platform for stool specimens. Stool samples were collected in Cary–Blair transport medium and processed according to the manufacturer’s instructions. The panel simultaneously detects 22 GI pathogens (bacteria, viruses, and parasites) from a single 0.2 mL aliquot, with results available in approximately 1 h. The assay provides binary positive/negative pathogen results based on proprietary cycle‐threshold criteria embedded in the assay software; quantitative Ct values are not reported for clinical interpretation. Reported performance of the GI panel shows high sensitivity (98.5%) and specificity (99.2%) across targets when compared with conventional methods. Internal controls for nucleic acid extraction and amplification were included as per the manufacturer’s protocol.

### 2.6. Statistical Analysis

Univariate descriptive analysis of demographic, clinical, treatment, and outcome variables was conducted. For categorical variables, absolute and relative frequencies were calculated. For quantitative variables, means and standard deviations or medians and interquartile ranges (IQRs) were used depending on the data distribution. The Kolmogorov–Smirnov test was used to assess normality.

A bivariate analysis was conducted to explore associations between the main outcome, diarrhea classification (acute, persistent, or chronic), and independent demographic, clinical, and outcome variables. Group comparisons were performed using Fisher’s exact test or chi‐square test for qualitative variables, and Kruskal–Wallis test or ANOVA test for quantitative variables, depending on their distribution. To compare diarrhea etiology (infectious vs. noninfectious), categorical variables were compared using the chi‐square test and are presented as proportions. Effect sizes are reported as odds ratios (ORs) with 95% CIs for binary outcome variables. Continuous variables are presented as medians with IQRs and compared using the Mann–Whitney *U* test. Statistical significance was set at *p* < 0.05. Statistical analyses were performed using Stata Version 16.

### 2.7. Ethical Considerations

The study was approved by the institutional review board (IRB) (approval No. 2131; minutes No. 07, April 5, 2023) and conducted in accordance with the Declaration of Helsinki and Colombian Resolution 8430 of 1993. Participant confidentiality was ensured through coded records and restricted access.

## 3. Results

A total of 84 patients were included, with a mean age of 42 years and a male‐to‐female ratio of 2.8:1. The most common comorbidities were cancer (33%), kidney disease (15.8%), and diabetes mellitus (15.5%). No significant differences in comorbidities were observed across the different diarrhea classifications (Table 1).

**TABLE 1:** Clinical characteristics, etiology, and outcomes in HIV‐positive patients according to diarrhea classification.

Characteristics	*n* = 84	Total	Diarrhea´s classification			*p* value
			Acute *n* = 63	Persistent *n* = 7	Chronic *n* = 14	
*Demographic characteristics*						

Female	84	22 (26.19)	20 (31.75)	1 (14.29)	1 (7.14)	0.166^c^
Male		62 (73.81)	43 (68.25)	6 (85.71)	13 (92.86)	
Age (years)^∗^	84	42 (14.7)	41 (24)	44 (29)	48 (23)	0.431^a^

*Comorbidities*						
Type 2 diabetes mellitus	84	13 (15.48)	7 (11.11)	2 (28.57)	4 (28.57)	0.122^c^
Cancer	84	28 (33.33)	24 (38.10)	2 (28.57)	2 (14.29)	0.230^c^
Chronic kidney disease	82	13 (15.85)	11 (18.03)	0 (0.00)	2 (14.29)	0.680^c^

*HIV-related characteristics*						
HIV viral load^∗∗^	84	344 (221,522)	160 (86,106)	400,423 (689,690)	21,750 (280,282)	**0.039** ^∗∗∗^
ART duration (months)^∗∗^	57	20.5 (53)	20.5 (52.5)	2 (2)	24 (18)	0.1152^∗∗∗^
CD4 (+) T‐cell count^∗∗^	81	301 (461)	353 (507)	39 (58)	273.5 (446)	**0.0044** ^∗∗∗^
Receiving ART	63	57 (90.48)	47 (92.16)	2 (66.67)	8 (88.89)	0.188^c^
De novo HIV diagnosis	84	21 (25.00)	12 (19.05)	4 (57.14)	5 (35.71)	0.046^c^
AIDS‐defining condition	84	20 (23.81)	10 (15.87)	5 (71.43)	5 (35.71)	**0.002** ^c^

*Clinical and laboratory findings*						
Vomiting	84	34 (40.48)	28 (44.44)	2 (28.57)	4 (28.57)	0.510^c^
Hematochezia/Enterorrheia	84	15 (17.86)	11 (17.46)	1 (14.29)	3 (21.43)	0.885^c^
Fever	84	44 (52.38)	33 (52.38)	5 (71.43)	6 (42.86)	0.490^c^
Number of bowel movements (per day)	67					
1–3		14 (20.9)	11 (21.5)	1 (20.0)	2 (18.2)	0.986^c^
4–6		33 (49.2)	25 (49.0)	2 (40.0)	6 (54.5)	
> 6		20 (29.9)	15 (29.4)	2 (40.0)	3 (27.3)	
Stool culture performed	84	36 (42.9)	26 (41.3)	4 (57.1)	6 (42.9)	0.752^c^
Molecular panel performed	84	19 (22.62)	11 (17.46)	3 (42.86)	5 (35.71)	0.123^c^
Etiologic diagnosis reached	84	43 (51.19)	30 (47.62)	4 (57.14)	9 (64.29)	0.569^c^
Pharmacological treatment for diarrhea	84	61 (72.6)	45 (71.4)	7 (100)	9 (64.3)	0.252^c^

*Clinical outcomes*						
ICU admission	84	19 (22.62)	13 (20.63)	4 (57.14)	2 (14.29)	0.077^c^
Length of hospital stay (days)^∗∗^	84	10 (14.5)	8.0 (15.0)	14.0 (36.0)	10.5 (14)	0.591^∗∗∗^
Diarrhea resolution	84	77 (91.7)	60 (95.3)	6 (85.7)	11 (78.6)	0.067^c^
Mortality	84	9 (10.7)	8 (12.7)	1 (14.3)	0 (0.0)	0.451^c^

^∗^Mean (SD).

^∗∗^Median (IQR).

^∗∗∗^Kruskal–Wallis.

^a^ANOVA.

^c^Fisher test.

**FIGURE 1:**
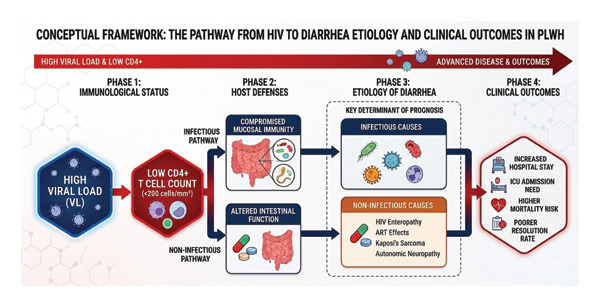
Conceptual framework: the pathway from HIV to diarrhea etiology and clinical outcomes in PLWH.

In terms of diarrhea characteristics, 75% of the cases were classified as acute, 8% as persistent, and 16% as chronic. A total of 49.2% of the patients presented 4–6 bowel movements per day. The associated symptoms included fever (52%), emesis (40%), and hematochezia/enterorrheia (18%), with no significant differences between diarrhea classification groups (Table 1) (see Figure 1).

Among the patients with a prior HIV diagnosis (75%), 90.5% were receiving ART. In this group, acute diarrhea was the most frequent presentation (92.2%), although this difference was not statistically significant (*p* = 0.188). The median ART duration was 20.5 months (IQR = 53 months). Patients with persistent diarrhea had a shorter duration of ART compared to the other groups, although this difference was not statistically significant (*p* = 0.1152). De novo HIV diagnoses accounted for 25% of all cases. The persistent diarrhea group showed a significantly higher proportion of de novo HIV diagnoses compared to those with acute or chronic diarrhea (*p* = 0.046) (Table 1).

Laboratory testing showed a median HIV viral load of 344 copies/mL (IQR: 221–522) and a median CD4(+) T‐cell count of 301 cells/μL (IQR: 461). Patients with persistent diarrhea had higher viral loads and lower CD4(+) T‐cell counts than those in the other groups (*p* = 0.039 and *p* = 0.004, respectively) (Table 1).

Overall, 22.6% of the patients required ICU admission. The median hospital stay was 10 days (IQR = 14.5 days). At the time of discharge, diarrhea resolved in 91.7% of cases. The overall mortality rate was 10.7% (*n* = 9 patients), with two deaths (2.8%) attributed to abdominal sepsis, one caused by cytomegalovirus (CMV) and the other by *E. coli* (Table 1).

Overall, 65.5% of the patients had infectious diarrhea. Analysis of the data by diarrhea type (infectious vs. noninfectious) revealed that patients with infectious diarrhea had significantly higher HIV viral loads and lower CD4(+) T‐cell counts compared to those with noninfectious diarrhea (*p* = 0.004 and *p* < 0.001, respectively). ICU admission occurred in 13/55 (23.6%) patients with infectious diarrhea and in 6/29 (20.7%) patients with noninfectious diarrhea. There was no significant difference between groups (OR: 1.18, 95% CI: 0.38–3.60) (Table 2).

**TABLE 2:** HIV characteristics and outcomes according to diarrhea etiology.

Characteristic	Infectious (*n* = 55)	Noninfectious (*n* = 29)	Effect size (95% CI)	*p* value
HIV viral load^†^	38,386 (343,917.5)	40 (260)	—	0.004
ART duration (months)^†^	12 (56)	24 (48)	—	0.092
CD4 (+) T‐cell count^†^	115.5 (382)	431 (457)	—	< 0.001
ICU admission	13 (23.6%)	6 (20.7%)	1.18 (0.39–3.53)	0.759
Length of hospital stay (days)^†^	8 (23)	10 (11)	—	0.770

*Note:* ICU admission: proportions compared using chi‐square test; odds ratios (ORs) with 95% confidence intervals (CIs).

^†^Median (IQR); comparisons using Mann–Whitney *U* test.

Regarding prior medication use, the most frequently reported drugs were statins (20.2%), angiotensin‐converting enzyme inhibitors (ACEIs) (17.9%), nonsteroidal anti‐inflammatory drugs (NSAIDs) (14.3%), and proton pump inhibitors (PPIs) (13.2%). Antimicrobial prophylaxis was reported in 7.1% of the patients, of whom 50% had received beta‐lactams (Supporting file 2). Nucleoside reverse transcriptase inhibitor (NRTI)–based regimens were the most common among patients receiving ART (Supporting file 3).

A total of 23.8% of patients presented with AIDS‐defining conditions, with Kaposi’s sarcoma (30%), esophageal or lower respiratory tract candidiasis (25%), and cerebral toxoplasmosis (20%) being the most common (Supporting file 4). These conditions were more prevalent among patients with persistent diarrhea, with a statistically significant difference (*p* = 0.002) (Table 1).

Statistically significant differences were observed in the mean AST and serum chloride levels across the three groups, with higher values in the persistent diarrhea group. ALT levels were also higher in this group than in patients with acute or chronic diarrhea, although this difference was not statistically significant (Supporting file 5).

With respect to imaging and endoscopic procedures, 33.3% of patients underwent colonoscopy, revealing macroscopic findings of colitis in 60.7%, ulcers in 39.3%, edema in 32.1%, and CMV identified histologically in 19.2% of cases. Abdominal computed tomography (CT) was performed in 46.4% of cases, identifying inflammation in 56.4%, and bowel perforation in 5.1% (Supporting file 6).

An etiologic diagnosis was established in 51.2% of the patients. Diagnostic testing was determined by clinical judgment, with stool culture being the most common method (42.9%), in which mixed microbiota was detected in 82% of cases. Multiplex molecular stool panels were used in 22.6% of the patients, identifying enteropathogenic *E. coli* (EPEC) as the most common pathogen (86.1%), followed by enteroinvasive *E. coli* (EIEC) (26%), and norovirus (15.8%). Additional diagnostic tests performed based on the medical criteria included histopathological detection of microorganisms, CMV viral load testing, and blood cultures (Supporting file 7).

Regarding treatment, 72.6% of patients required pharmacological management during hospitalization (Table 1), with antibiotics being the most frequently used therapy (57.1%) (Supporting file 7). Of these patients, 23 received antibiotic treatment for documented bacterial infections other than diarrhea. Eleven patients were treated specifically for diarrhea in accordance with the institutional protocol “Infectious Etiology Diarrhea–GM‐FVL‐XXX,” which defines the indications for initiating empirical therapy and subsequent targeted treatment. Eight patients received antibiotics for two indications: prophylaxis based on their CD4+ T‐lymphocyte count and management of diarrhea, while six patients initiated antibiotic prophylaxis alone. As all patients were hospitalized at the institution, antimicrobial therapy was reviewed 48 h after initiation in all cases, and all treatment regimens were consistent with institutional management protocols.

## 4. Discussion

Regarding demographic characteristics, diarrhea in PLWH occurred more frequently in men, with a mean age of 42 years. These findings are consistent with the literature, which reported a higher incidence in males during the fifth decade of life [5].

Cancer was the most prevalent comorbidity, followed by chronic kidney disease and diabetes mellitus. Notably, when comparing these rates with those reported in PLWH without diarrhea, diabetes occurs in only approximately 3% of the population and cancer in 0.4% of the population [6]. This higher prevalence could be attributed to the complexity of patients treated at our institution, which is a tertiary referral center, and to the increased risk of malignancies in PLWH, even in those receiving effective ART [7]. Factors contributing to this elevated cancer risk include HIV‐induced immunosuppression, co‐infection with oncogenic viruses such as human papillomavirus (HPV) and Human herpesvirus 8 (HHV‐8), and behavioral factors such as smoking [8].

In our study, 25% of the patients with diarrhea had a de novo HIV diagnosis. This is lower than the rates reported in the literature, where 40%–80% of ART‐naïve patients present with diarrhea [9]. However, among patients with persistent diarrhea, a higher proportion were newly diagnosed with HIV, highlighting the importance of considering HIV testing in patients presenting with diarrhea, even when the duration is not chronic.

A total of 23% of the patients presented with AIDS‐defining conditions, most commonly Kaposi’s sarcoma and esophageal candidiasis. A Colombian study reported a prevalence of 33% for opportunistic infections in PLWH, with *tuberculosis*, histoplasmosis, and cryptococcosis being the most frequent etiologies [5]. Our findings regarding Kaposi’s sarcoma align with other Colombian studies showing that 87% of Kaposi’s sarcoma cases occur in patients with HIV co‐infection [10].

More than 90% of patients with previously diagnosed HIV were receiving ART, which is higher than the 62% reported in other Colombian studies [5] and the 77% reported globally [11]. ART regimens in this cohort were aligned with the current first‐line recommendations, mainly composed of NRTIs and integrase inhibitors [1].

Most diarrhea episodes were acute, consistent with findings from other Colombian studies [12]. Stool microscopy was the most frequently used initial diagnostic test, in line with recommendations from the literature regarding its utility and cost‐effectiveness [13]. Microbiological identification using stool culture and molecular panels yielded a diagnosis in 51% of cases, which is comparable to the 50% reported in the literature [5].

Molecular diagnostics proved to be superior to conventional stool cultures, with 100% sensitivity and 97% specificity. In the present cohort, the etiology of infectious diarrhea was dominated by bacterial pathogens, particularly EPEC. This finding is consistent with the study by Salazar‐Arenas et al., which evaluated the microbiological characteristics of patients with diarrhea using a GI panel in a Colombian cohort. In that context, the high frequency of EPEC did not differ according to the presence of immunosuppression [14]. Similarly, the study by Montalvo‐Otivo et al. also identified EPEC as the most prevalent pathogen [15]. This consistent regional predominance supports EPEC as a leading cause of infectious diarrhea in Latin America, regardless of immune status.

Notable differences were observed in the diversity of diarrheagenic *E. coli* pathotypes compared to previous studies. Montalvo‐Otivo et al. reported substantial frequencies of enteroaggregative (EAEC) (40%), enterotoxigenic (ETEC) (29%), and Shiga toxin–producing *E. coli* (STEC) (11%), whereas these pathotypes were absent or infrequent in our cohort. In contrast, the Colombian cohort of Salazar‐Arenas et al. demonstrated intermediate prevalence rates of EAEC (18.6%), ETEC (7%), and STEC (5.2%), with higher frequencies among immunosuppressed patients compared to nonimmunosuppressed individuals. Other invasive bacterial pathogens also showed differential patterns, with *Shigella*/*EIEC* being more frequent in our cohort than the Mexican study (15%) and the previous Colombian cohort (11.6%), particularly among immunosuppressed patients [14, 15]. These findings suggest that while EPEC predominates regionally, the distribution of other bacterial pathogens varies according to population characteristics, immune status, and possibly environmental exposure.

Norovirus was the most frequently identified virus across all the regional studies; however, its prevalence was higher in the previous Mexican and Colombian cohorts compared to the present findings [14, 15]. This difference may be related to the predominance of bacterial etiologies and empiric antibiotic use in hospitalized patients with advanced immunosuppression. Other viruses, including rotavirus, astrovirus, and sapovirus, were detected at low frequencies, with minimal contribution in the present study.

Regarding parasitic infections, prevalence rates were low, similar to those reported in other regional studies [14, 15], with slight variability depending on the parasite species, which may be related to local sanitation strategies, personal hygiene practices, food handling, and other contextual factors. Within the Colombian study, protozoal infections were more frequent among immunosuppressed individuals [14], aligning with the known susceptibility of these patients to opportunistic enteric parasites.

The predominance of bacterial pathogens such as EPEC and the relatively low prevalence of opportunistic enteric parasites observed in our cohort may reflect, at least in part, the impact of ART scale‐up in the region. Similar to findings reported by regional studies [14, 15], the etiological profile in our population appears to resemble that of the general population rather than the classical spectrum of opportunistic infections historically described in advanced HIV disease. This shift in pathogen ranking suggests that widespread ART use may have modified the epidemiology of infectious diarrhea among PLWH, with a reduced contribution of opportunistic parasites and a greater relative importance of common bacterial and viral enteropathogens.

Endoscopic evaluation was also performed and is recommended for both etiologic investigation and assessment of comorbidities and complications in these patients [16]. CMV was the most common pathogen identified on histopathological examination, which is consistent with prior literature on endoscopic findings in this population [17, 18].

Despite the high ART coverage in our cohort, infectious diarrhea remained predominant (65.5%). This contrasts with the findings of Dikman et al. and Call et al., who reported a shift toward noninfectious etiologies in the ART era [2, 3]. Furthermore, persistent diarrhea and infectious etiology were significantly associated with more advanced stages of HIV infection, as evidenced by higher viral loads (*p* = 0.039 and 0.004, respectively) and lower CD4(+) T‐cell counts (*p* = 0.004 and 0.001, respectively), consistent with previous literature. The association between persistent diarrhea and advanced HIV remains underexplored, as most studies have focused on chronic diarrhea rather than persistent or acute presentations [15, 16].

Laboratory tests revealed elevated liver enzyme levels in patients with persistent diarrhea along with higher viral loads. Previous studies have suggested that elevated aminotransferases in PLWH may be due to hepatotropic viral co‐infections, ART‐induced hepatotoxicity, or direct hepatocyte damage mediated by HIV. In the latter scenario, the presence of HIV, reflected in a detectable viral load, can induce hepatocyte apoptosis via caspase activation, mitochondrial dysfunction, and altered membrane permeability triggered by viral proteins [19, 20].

Lastly, our study reported higher ICU admission rates (22% vs. 12%) compared to another Colombian study by Montúfar et al. [5]. This may reflect the complexity of the cases treated at our tertiary care center, which has greater ICU availability and access. However, the mortality rates were not significantly higher.

## 5. Conclusions

Despite increased access to ART, infectious etiologies continue to predominate in cases of diarrhea among PLWH in our setting. Most patients experienced resolution of symptoms, indicating that although infections persist, treatment remains effective in the majority of cases.

Traditionally, chronic diarrhea has been regarded as a hallmark of HIV infection. However, the findings from this study suggest that HIV should also be considered in the differential diagnosis of patients presenting with diarrhea of shorter duration.

Patients with higher HIV viral loads and lower CD4(+) T‐cell counts were significantly more likely to present with diarrheal episodes, particularly the persistent type. These individuals also showed a trend toward longer hospital stays, increased ICU admissions, and higher mortality rates, although the latter was not statistically significant.

Further research is needed to better understand the outcomes associated with specific pathogens identified in HIV‐positive patients with diarrhea. A prospective cohort using standardized diagnostics and 30‐day follow‐up is required to quantify attributable mortality and validate these risk factors, optimizing the clinical management and preventing further complications in this population.

### 5.1. Strengths and Limitations

• This study provides relevant information regarding the causes of diarrhea in PLWH, highlighting the notable prevalence of infectious etiologies, such as EPEC and norovirus. These findings may help improve therapeutic and preventive strategies.
• Despite being conducted in a high‐complexity institution, stool microscopy is the most commonly used diagnostic method. This aligns with the current recommendations and represents an accessible initial approach for diarrhea assessment in lower‐complexity healthcare settings.
• As a cross‐sectional study, it does not allow for the establishment of causal relationships between variables.
• Microbiological isolation and diagnostic methods (e.g., stool cultures and molecular panels) were selected based on the clinician’s judgment, which may have introduced variability in the results.
• The study population was drawn from a tertiary care center, where medical decisions were made by specialists in infectious diseases and gastroenterology. This limits the generalizability of the findings to lower‐complexity healthcare facilities such as Level I or II hospitals.

## Funding

No funding was received for this study.

## Conflicts of Interest

The authors declare no conflicts of interest.

## Supporting Information

Additional supporting information can be found online in the Supporting Information section.

## Supporting information


**Supporting Information** Supporting file 1. Variables and definitions. Supporting file 2. Pharmacological history. Supporting file 3. Antiretroviral treatment regimen. Supporting file 4. AIDS‐defining conditions. Supporting file 5. Initial laboratory tests performed in patients with diarrhea and HIV. Supporting file 6. Endoscopic and imaging studies. Supporting file 7. Microbiological testing and pharmacological management of diarrhea.

## Data Availability

The data that support the findings of this study are available on request from the corresponding author. The data are not publicly available due to privacy or ethical restrictions.
