# Supplementary material for: HIV and Diarrhea: Clinical Characteristics and Etiology at a High‐Complexity Center in Southwest Colombia
Source: AIDS Res Treat. 2026 Jun 23;2026:7874615. doi: 10.1155/arat/7874615 (PMC13291440; doi:10.1155/arat/7874615)
Supplement: Supplementary file 1 — Supporting Information Supporting file 1. Variables and definitions. Supporting file 2. Pharmacological history. Supporting file 3. Antiretroviral treatment regimen. Supporting file 4. AIDS‐defining conditions. Supporting file 5. Initial laboratory tests performed in patients with diarrhea and HIV. Supporting file 6. Endoscopic and imaging studies. Supporting file 7. Microbiological testing and pharmacological management of diarrhea. [file ARAT-2026-7874615-s001.docx]

**Supplementary file 1. Variables and definitions**

| **Variable** | **Operational definition** |
| --- | --- |
| Diarrhea | Presence of ≥3 loose or watery stools per day, documented in the medical record. |
| Duration of diarrhea | Classified as acute (≤14 days), persistent (15–28 days), or chronic (>28 days). |
| Infectious diarrhea | Diarrhea with at least one of the following: (1) positive stool culture or PCR for enteric pathogens; or (2) initiation of antimicrobial therapy with documented clinical response within 72 hours. |
| Non-infectious diarrhea | Diarrhea with negative infectious work-up and no antimicrobial therapy, or antimicrobials discontinued within 24 hours, with a documented alternative non-infectious cause (e.g., antiretroviral therapy, malignancy, inflammatory or functional etiology). |
| Receiving ART | Use of antiretroviral therapy at admission. Record whether regimen contained a protease-inhibitor |
| ART duration (months) | Interval between ART initiation and hospital admission, expressed in months. |
| AIDS-defining conditions | Presence of opportunistic infections or malignancies indicative of advanced HIV infection, including esophageal candidiasis, Pneumocystis jirovecii pneumonia, and Kaposi’s sarcoma. |
| Antimicrobial prophylaxis | Use of azithromycin, trimethoprim/sulfamethoxazole, ganciclovir, valganciclovir, or fluconazole prior to hospital admission. |
| Clinical events | In-hospital mortality, length of hospital stay, need for intensive care unit admission, and clinical response to treatment as documented in the medical record. |

**Supplementary file 2. Pharmacological History**

| **Variable** | **N** | **n (%)** |
| --- | --- | --- |
| Statins | 84 | 17 (20.23) |
| ACE Inhibitors (ACEIs) | 84 | 15 (17.9) |
| NSAIDs | 84 | 12 (14.3) |
| Proton Pump Inhibitors (PPIs) | 83 | 11 (13.2) |
| Metformin | 84 | 11 (13.1) |
| Psychiatric medications          SSRIs | 84  9 | 9 (10.7)  5 (55.6) |
| Antimicrobials          Beta-lactams | 84  6 | 6 (7.1)  3 (50) |

**Supplementary file 3. Antiretroviral Treatment Regimens**

| **Variable** | **N** | **n (%)** |
| --- | --- | --- |
| **NRTIs** | 57 | 53 (93) |
| Emtricitabine (FTC) | 53 | 29 (54.7) |
| Tenofovir disoproxil fumarate (TDF) | 53 | 28 (52.8) |
| Lamivudine (3TC) | 53 | 18 (34.0) |
| **Integrase Inhibitors** | 57 | 40 (70.2) |
| Dolutegravir (DTG) | 40 | 34 (85) |
| Raltegravir (RAL) | 40 | 5 (12.5) |
| Elvitegravir (EVG) | 40 | 1 (2.5) |
| **Protease Inhibitors** | 57 | 12 (21.0) |
| Ritonavir (RTV) | 12 | 9 (75) |
| Darunavir (DRV) | 12 | 7 ( 58.3) |
| Atazanavir (ATV) | 12 | 4 (33.3) |
| **NNRTIs** | 57 | 11 (19.3) |
| Efavirenz (EFV) | 11 | 7 (63.6) |
| Nevirapina (NVP) | 11 | 2 (18.2) |
| Rilpivirina (RVP) | 11 | 1 (9.1) |

**Supplementary file 4. AIDS-Defining Conditions**

| **Variable** | **N** | **n (%)** |
| --- | --- | --- |
| **Presence of AIDS-defining condition** | 84 | 20 (23.8) |
| Kaposi’s sarcoma | 20 | 6 (30.0) |
| Esophageal, bronchial, tracheal, or pulmonary candidiasis (excluding oral) | 20 | 5 (25.0) |
| Cytomegalovirus CMV | 20 | 5 (5.90) |
| Cerebral  toxoplasmosis | 20 | 4 (20.0) |
| Tuberculosis | 20 | 3 (15.0) |
| Immunoblastic lymphoma | 20 | 2 (10.0) |

**Supplementary file 5. Initial Laboratory Tests Performed in Patients with Diarrhea and HIV**

| **Parameter** | **n = 84** | **Total** | **Diarrhea´s classification** | | | **p-value** |
| --- | --- | --- | --- | --- | --- | --- |
|  |  |  | **Acute**  **n = 63** | **Persistent n=7** | **Chronic**  **n = 14** |  |
| **Laboratory Tests** | | | | | | |
| White blood cells* | 84 | 6786.7 (4395) | 6788.2 (4577.6) | 6741.4 (4982.0) | 6802.1 (3454.0) | 0.665Ú |
| Neutrophils** | 84 | 3620 (4005) | 3570 (4170) | 4179 (4800) | 4150 (3700) | 0.9187& |
| Lymphocytes* | 84 | 1393.3 (922) | 1336,6 (888,0) | 1280 (780,2) | 1705 (1121,9) | 0.611Ú |
| Hemoglobin** | 84 | 12.0 (4,7) | 12 (4,8) | 13 (4,7) | 12.9 (4,3) | 0,814& |
| Platelets** | 84 | 237500 (111500) | 233000 (136000) | 231000 (231000) | 268000 (112000) | 0,276& |
| C-reactive protein** | 84 | 4.5 (14.2) | 5.3 (17.1) | 9.9 (12,3) | 1,35 (4,4) | 0.1389& |
| Serum sodium* | 82 | 135.7 (5.8) | 135,5 (5,0) | 138 (10,8) | 135,5 (6,8) | 0,239Ú |
| Serum potassium* | 82 | 4.0 (0.5) | 4,0 (0,5) | 4,1 (0,6) | 4,0 (0,5) | 0,946Ú |
| Serum magnesium* | 55 | 1,8 (0.4) | 1,9 (1,06) | 2.1 (0,6) | 1,8 (0,3) | 0,947Ú |
| Serum chloride * | 69 | 101 (6,6) | 101.1 (5.7) | 104.5 (6.8) | 100.8 (9.9) | **0,006Ú** |
| Aspartate aminotransferase (AST)** | 68 | 26.1 (36.7) | 23,3 (24,2) | 70,8 (106,7) | 35,9 (61,1) | **0,0244&** |
| Alanine aminotransferase (ALT) ** | 68 | 25.5 (44.4) | 21,9 (27.0) | 65,7 (50,0) | 17 (66,6) | 0,0893& |
| **Median (IQR) *Mean (SD)  & Krustal Wallis  Ú Anova  ↟ Chi squared Test    ^ Fisher Test | | | | | | |

**Supplementary file 6. Endoscopic and Imaging Studies**

| **Variable** | **N** | **n (%)** |
| --- | --- | --- |
| **Colonoscopy performed** | 84 | 28 (33.3) |
| **Colitis** | 28 | 17 (60.7) |
| **Other finding:** |  |  |
| Normal | 28 | 10 (35.7) |
| Ulcers | 28 | 11 (39.3) |
| Edema | 28 | 9 (32.1) |
| Erythema | 28 | 8 (28.6) |
| **Abdominal CT scan** | 84 | 39 (46.4) |
| Evidence of perforation | 39 | 2 (5.1) |
| Evidence of intestinal inflammation | 39 | 22(56.4) |

**Supplementary file 7. Microbiological Testing and Pharmacological Management of Diarrhea**

| **Variable** | **N** | **n (%)** |
| --- | --- | --- |
| **Stool microscopy performed** | 84 | 53 (63.1) |
| Occult blood positive | 53 | 43 (81.1) |
| >8 leukocytes per field | 53 | 24 (45.3) |
| **Stool culture performed** | 84 | 36 (42.9) |
| Mixed microbiota | 36 | 31 (86.1) |
| Other microorganisms | 36 | 5 (13.9) |
| **Gastrointestinal molecular panel performed** | 84 | 19 (22.6) |
| Enteropathogenic *E. coli (EPEC)* | 19 | 11 (57.9) |
| *Shigella/*Entero-invasive *E. coli (EIEC)* | 19 | 5 (26.3) |
| Norovirus | 19 | 3 (15.8) |
| **Blood Culture performed** | 84 | 46 (54.7) |
| *Escherichia coli* | 46 | 2 (4.3) |
| *Pseudomonas aeruginosa* | 46 | 2 (4.3) |
| *Streptococcus dysgalactiae* | 46 | 2 (4.3) |
| **Cytomegalovirus Viral Load** | 84 | 11 (13.1) |
| **Histopathological Findings** | 28 | 26 (92.9) |
| Histoplasma | 26 | 1 (3.8) |
| Cytomegalovirus | 26 | 5 (19.2) |
| **Pharmacological management of diarrhea** |  |  |
| Antibiotic | 84 | 48 (57.1) |
| Antidiarrheal agents | 84 | 9 (10.7) |
| Combination therapy | 84 | 4 (4.8) |
